# Circulating MiRNAs of ‘Asian Indian Phenotype’ Identified in Subjects with Impaired Glucose Tolerance and Patients with Type 2 Diabetes

**DOI:** 10.1371/journal.pone.0128372

**Published:** 2015-05-28

**Authors:** Paramasivam Prabu, Sophie Rome, Chandrakumar Sathishkumar, Sankaramoorthy Aravind, Balakumar Mahalingam, Coimbatore Subramanian Shanthirani, Caroline Gastebois, Audrey Villard, Viswanathan Mohan, Muthuswamy Balasubramanyam

**Affiliations:** 1 Madras Diabetes Research Foundation and Dr. Mohan’s Diabetes Specialities Centre, WHO Collaborating Centre for Non-Communicable Diseases Prevention and Control & IDF Centre of Education, Gopalapuram, Chennai-600086, India; 2 CarMeN Laboratory (INSERM 1060, INRA 1397, INSA), University of Lyon, Faculté de Médecine Lyon-Sud, Chemin du Grand Revoyet, 69600, Oullins, France; SAINT LOUIS UNIVERSITY, UNITED STATES

## Abstract

Several omics technologies are underway worldwide with an aim to unravel the pathophysiology of a complex phenotype such as type 2 diabetes mellitus (T2DM). While recent studies imply a clinically relevant and potential biomarker role of circulatory miRNAs in the etiology of T2DM, there is lack of data on this aspect in Indians—an ethnic population characterized to represent ‘Asian Indian phenotype’ known to be more prone to develop T2DM and cardiovascular disease than Europeans. We performed global serum miRNA profiling and the validation of candidate miRNAs by qRT-PCR in a cohort of subjects comprised of normal glucose tolerance (NGT), impaired glucose tolerance (IGT) and patients with T2DM. Our study revealed 4 differentially expressed miRNAs (miR-128, miR-130b-3p, miR-374a-5p, miR-423-5p) in subjects with IGT and T2DM patients compared to control subjects. They were positively or negatively correlated to cholesterol levels, HbA_1C_, HOMA-IR and fasting insulin. Interestingly, circulating level of miR-128 and miR-130b-3p were also altered in serum of diet-induced diabetic mice compared to control animals. Among the altered circulating miRNAs, miR-128 had never been described in previous studies/populations and appeared to be a ‘New Lead’ in Indians. It was positively correlated with cholesterol both in prediabetic subjects and in diet-induced diabetic mice, suggesting that its increased level might be associated with the development of dyslipedemia associated with T2DM. Our findings imply directionality towards biomarker potential of miRNAs in the prevention/diagnosis/treatment outcomes of diabetes.

## Introduction

South Asia forms one of the epicenters of the global diabetes epidemic. Over the past couple of decades, there has been a worrying increase in the prevalence rates of type 2 diabetes mellitus (T2DM) in the region and India in particular. The higher prevalence of diabetes in South Asians is least explained by traditionally measured risk factors. Insulin resistance and abnormalities of insulin secretion in pancreatic ß-cells are the main defects that lead to T2DM. The so called “Asian Indian Phenotype” refers to certain unique clinical and biochemical abnormalities in Indians which include increased insulin resistance, greater abdominal adiposity *i*.*e*., higher waist circumference despite lower body mass index and T2DM occurring at a younger age than Caucasians [1,2]. South Asians are also characterized by a unique metabolic profile with higher insulin levels [3], a greater degree of insulin resistance [4] and a higher prevalence of diabetes [5]. Insulin resistance has been demonstrated in Asian Indians even during adolescence [6]and hyperinsulinemia seems to be present in Asian Indians even at birth [7]. More recent studies do imply that South Asians may have an early decline in ß-cell functions as well [8,9,2].

Given the predicted explosion in the number of cases of prediabetes and T2DM worldwide and in India, continued research is essential, particularly in the newer areas such as application of microRNA (miRNA) technologies. MiRNAs are a class of evolutionally conserved non-coding RNAs of 19–22 nucleotides and function as negative regulators of gene expression [10]. Important roles of miRNAs have emerged in the control of metabolic pathways involved in lipid metabolism, adipocyte differentiation and pancreas development, energy homeostasis, glucose-stimulated insulin secretion and inflammation [11–16]. Recently, significant amounts of miRNAs have been found not only intracellularly, but in extracellular human body fluids (*e*.*g*.; serum, plasma, saliva, urine, tears, amniotic fluids and milk) [17–19]. They are remarkably stable despite high extracellular RNAse activities. Extracellular miRNAs are enclosed in small membranous vesicles (*e*.*g*.; in exosomes, shedding vesicles, apoptotic bodies) or associated with, or packaged within high-density lipoprotein, or associated with RNA-binding proteins (*e*.*g*.; high-density lipoprotein, Argonaute 2 and nucleophosmin 1) [20–22]. Levels of miRNAs in the serum of humans have been shown to be stable, reproducible, consistent amongst healthy individuals and change during pathophysiology, allowing them to be of potential value as biomarkers of disease [23]. Recently, novel circulatory miRNA signatures have been studied and shown associated not only with the disease but also the severity of diseases such as various cancers, liver and cardiovascular diseases [24,25]. Whereas several studies have evaluated the role of miRNAs in cancer, much less is known in the field of diabetes. Differentially expressed circulatory miRNA signature characteristic of T2DM have been reported from recent studies outside the India based on the analysis of small group of subjects [26–33]. From these studies, it is expected that the identification of potential-specific biomarkers of miRNAs may help predict or detect the development and progression of diabetes and its complications at an early stage, and therefore allow timely intervention. However, there is lack of clinically relevant data on the potential of circulatory miRNAs in Indians, who are highly insulin resistant and more prone to develop T2DM and cardiovascular disease than Europeans [5]. While the health burden of T2DM is somehow associated with obesity as a co-morbidity in Caucasians, metabolic risk factors are either higher in Asian Indians independent of obesity or operate at a lower BMI threshold. Therefore, this study is aimed to identify circulating miRNAs that could be differentially expressed in subjects with prediabetes and patients with T2DM and to dissect out their potential biomarkers role in association with clinical parameters/conventional risk factors of T2DM.

## Research Design and Methods

### Recruitment of the Study Subjects

Subjects with NGT (Normal Glucose Tolerance, n = 49), subjects with IGT (Impaired Glucose Tolerance, n = 47) and patients with Type 2 Diabetes Mellitus (T2DM, n = 49) were recruited from Dr.Mohans’ Diabetes Specialities Centre, Chennai and from the on-going epidemilogical studies. The study was conducted according to the principles of Declaration of Helsinki and appropriate approval by the Institutional Ethics Committee of the Madras Diabetes Research Foundation. Ethics Committee approved written informed consent was obtained from all study subjects. NGT, IGT and T2DM were defined using World Health Organization consulting group criteria. Those who were confirmed by oral glucose tolerance test to have 2-hour plasma glucose value 11.1 mmol/L (200 mg/dL) or more based on World Health Organization consulting group criteria were diagnosed as diabetic patients. Those with 2-hour post glucose value ≥7.8 mmol/L (140 mg/dL) and <11.1 mmol/L (200 mg/dL) were diagnosed as subjects with IGT, and those with 2-hour post glucose value of less than 7.8 mmol/L (140 mg/dL) as subjects with NGT. To rule out the medication effects, all our T2DM subjects are of newly diagnosed nature.

### Anthropometric Measurements

Anthropometric measurements including height, weight and waist circumstance were obtained using standardized techniques. Height was noted with a tape measured to the nearest centimeter. Weight was measured with traditional spring balance that was kept on a firm horizontal surface. The body mass index (BMI) was calculated using the formula, weight (kg)/height (m)^2^. Waist circumference was measured using a non stretchable fiber measuring tape. Blood pressure was recorded from the right arm of study subjects when they were relaxed and in sitting position to the nearest 2mm Hg with a mercury sphygmomanometer (Diamond Deluxe BP apparatus, Pune, India). Two reading were taken 5 min apart and the mean of the two was taken as the blood pressure.

### Biochemical Parameter Investigations

Fasting plasma glucose (glucose oxidase-peroxidase method), serum cholesterol (cholesterol oxidase-peroxidase-amidopyrine method), serum triglycerides (glycerol phosphate oxidase-peroxidase-amidopyrine method) and HDL cholesterol (direct method-polyethylene glycol-pretreated enzymes) were measured using Hitachi-912 Autoanalyser (Hitachi, Mannheim, Germany). The intra and inter assay co-efficient of variation for the biochemical assays were <5%. Low-density lipoprotein (LDL) cholesterol was calculated using Friedewald formula. Glycated hemoglobin (HbAlc) was estimated by high-pressure liquid chromatography using the variant analyzer (Bio-Rad, Hercules, Calif., USA). Serum Insulin was estimated using enzyme-linked immunosorbent assay (Calbiotech, CA). The intra-assay and the inter-assay coefficients of variation for insulin assay was <10%. Insulin resistance was calculated using the homeostasis assessment model (HOMA-IR) using the formula: {fasting insulin (μIU/mL) x fasting glucose (mmol/L)} / 22.5.

### Circulating RNA Extraction and Purification

Fasting serum sample (from 5 ml of blood) was obtained by standard venepuncture using VacutainerPlus Plastic Serum and SST Tubes (Becton-Dickinson, Franklin, lakes, NJ). The separation of the serum was performed by centrifugation at 4,000 rpm for 10 min, followed by 12000 rpm for 15 min to completely remove cell debris. Total RNA was isolated from 0.25 ml of serum using Qiagen miRNeasy Mini kit (Qiagen, Valencia, CA) for RNA collection and purification according to the manufacturer's protocol. Total RNA was controlled using Nanodrop 2000 UV spectrophotometer and stored at -80°C until use.

### Global Serum microRNA Profiling

Global serum miRNA profiling was done in a discovery cohort comprised of 12 individuals each of NGT, IGT and T2DM **(Table 1)**. To assess hemolysis two microRNAs were used. One that is expressed in red blood cells (miRNA-451), and one that is relatively stable in serum and plasma and not affected by hemolysis (miRNA-23a). The ratio between these two miRNAs correlates to degree of hemolysis. In our experience samples with ratios above 8.0 will have an increased risk of being affected by hemolysis. We validated that our samples had lower ratios and were not affected by hemolysis. Due to the low levels of microRNAs and potentially high levels of inhibitors in samples derived from serum/plasma which also differs from sample to sample, Exiqon protocol (www.exiqon.com/serum-plasma-guidelines) recommends using RNA amounts based on starting volume rather than RNA quantity. 8 μL of eluted human plasma/serum RNA in a 40 μL RT reaction generally give a good signal with maximal miRNA detection in miRNA PCR panels [34]. Therefore in our study, 8 μl RNA was reverse transcribed in 40μl reactions using the miRCURY LNA Universal RT microRNA PCR cDNA synthesis kit. An RNA spike-in control (Sp6) was added to the reverse transcription step. This control is used to confirm that the reverse transcription and amplification occurs with equal efficiency in all samples. cDNA was diluted 50x and assayed in 10 μl PCR reactions according to the protocol for miRCURY LNAUniversal RT microRNA PCR; each microRNA was assayed once by qPCR on the microRNA Ready-to-Use PCR (miRCURY LNA microRNA Human panel I Exiqon). Negative controls excluding template from the reverse transcription reaction was performed and profiled like the samples. The amplification was performed in a LightCycler 480 Real-Time PCR System (Roche) in 384 well plates. The amplification curves were analyzed using the Roche LC software, both for determination of Cp (by the 2nd derivative method) and for melting curve analysis. Amplification efficiency was calculated using algorithms similar to the LinReg software. All assays were inspected for distinct melting curves and the Tm was checked to be within known specifications for the assay. Furthermore assays must be detected with 5 Cp’s less than the negative control, and with Cp<37 to be included in the data analysis. Data that did not pass these criteria were omitted from any further analysis. Using NormFinder the best normalization procedure was found to be the average of assays detected in all samples. All data were normalized to the average of assays detected in all samples (average—assay Cp). Comparison between groups were made by using the student *t*-test (p<0.05) on normalized data, to select the differentially expressed miRNAs.

**Table 1:** Clinical and biochemical characteristics of the subjects involved in the study.

	Whole cohort			Subgroup of subjects used to select candidate miRNAs		
Parameters	NGT	IGT	T2DM	NGT	IGT	T2DM
	(n = 49)	(n = 47)	(n = 49)	(n = 12)	(n = 12)	(n = 12)
Males/females	(26/23)	(23/24)	(25/24)	(6/6)	(6/6)	(5/7)
Age(years)	44.3 ± 6.9	44.1 ± 7.0	44.4 ± 8.1	38.5 ± 3.0	41.2 ± 4.0	40.2 ± 5.0
BMI(kg/m2)	24.5 ± 2.6	24.9 ± 2.9	25.7 ± 3.5	23.1 ± 2.0	23.9 ± 1.0	26.7 ± 1.0
Fasting plasma glucose (mg/dl)	85 ± 11	**105**± **18** *	**146** ± **34** *	83 ± 7.0	**101** ± **7.0** *	**150** ± **27** *
2 hr plasma glucose (mg/dl)	96 ± 21	**166** ± **18** *	**269** ± **63** *	89 ± 24	**160** ± **20** *	**264** ± **61** *
HbA1c (%)	5.6 ± 0.4	**6.3** ± **0.8** *	**7.8** ± **1.6** *	5.2 ± 0.5	6.0 ± 0.5	**7.9** ±± **1.0** *
HOMA-IR	2.0 ± 1.1	**3.5** ± **1.4** *	**6.3 ± 1.8** *	1.5 ± 0.4	2.7 ± 1.0	**5.0** ± **3.0** *
Serum Cholesterol (mg/dl)	170 ± 24	187 ± 35	185 ± 52	168 ± 29	185 ± 39	185 ± 52
Serum Triglycerides (mg/dl)	97 ± 38	148 ± 76	136 ± 62	119 ± 65	134 ± 69	148 ± 67
HDL cholesterol (mg/dl)	41.2 ± 9.0	40 ±5.0	37 ± 7.0	41 ± 9.0	41 ± 10	40 ± 8.0
LDL cholesterol (mg/dl)	111 ± 22	117 ± 32	121 ± 46	107 ± 26	117 ± 33	115 ± 44
VLDL (mg/dl)	19 ± 7.6	30 ± 16	25 ± 13	24 ± 13	27 ± 14	29 ± 13
Fasting Insulin (μIU/ml)	7.6 ± 2.0	11 ± 4.0	13 ± 6.7	9.6 ± 4.6	**13.4** ± **4.6** *	**17.8** ± **4.0** *
Systolic blood pressure (mmHg)	117 ± 9.0	121 ± 12	124 ± 12	123 ± 20	124 ± 12	126 ± 16
Diastolic blood pressure (mmHg)	78 ± 10	82 ± 5.0	81 ± 7.0	80 ± 10	80 ± 8.0	84 ± 9
Waist circumference (cm)	87.1 ± 1.21	**93.8** ± **1.60** *	**92.3** ± **1.38** *	87.3 ± 8.4	**93.5** ± **10.9** *	**92.3 ± 9.6** *

All the value represents mean and ± standard deviation

*p<0.05 compared control

ND, not determined

### Validation of candidate miRNAs by individual qRT-PCR

miRNAs short listed as differentially expressed from the discovery set were revalidated in a larger cohort comprising of 145 individuals (50% men, 50% women) with NGT (n = 49), IGT (n = 47) and T2DM (n = 49) by individual PCR assays. Total RNA from serum was extracted as described above and used for qRT-PCR by using the Universal miRCURY LNA microRNA PCR, Polyadenylation and cDNA synthesis kit II and the miRCURY LNA microRNA PCR system, Exilent SYBR green master mix, from Exiqon, on ABI 7000 Applied Biosystems thermocycler. Relative expression of microRNA expression was calculated by 2^ˆ(-"DeltaCt")^ method. We evaluated a suitable number of reference miRNAs, based on the increased expression stability [28]. However, we were unable to identify stable miRNAs that did not discriminate between male and female or that were not correlated with metabolic parameters, or that was not previously identified as relevant for the pathology in previous studies. This could be explained partly by the fact that inside the same group, women and men might differed in their level of cholesterol and triglycerides (S1 Table). Thus, in this study, data were normalised on the volume of total RNA that was used for qRT-PCR (*i*.*e*.; a fixed volume of 8 μl).

### Statistical Methods

Statistical analyses were performed with the SPSS statistical software (SPSS V12.0, Inc., Chicago, IL), and the R Statistical Software (http://www.r-project.org/). ANOVA and/or paired *t*-tests were performed to study differences on quantitative variables between groups.

## Bioinformatic Analysis

Cellular pathway determination for each miRNA was done by using DIANA-miRPath (http://diana.imis.athena-innovation.gr/DianaTools/index.php?r=site/index) which predicted target genes.

### Animals Maintenance, Preclinical Characterization and miRNA Profiling

Male C57BL/6J mice, 10–12 weeks old (body weight, 18–22 g) were obtained from Sri Venkateswara Enterprises (Bangalore) and maintained at 22±1°C under a 12-h light-dark cycle (lights on from 6:00 AM to 6:00 PM). All experiments were performed in accordance with regulations specified by the Committee for the Purpose of Control and Supervision on Experiments on Animals (CPCSEA), Government of India and approval by the Institutional Animal Ethical Committee (IAEC) of the Madras Diabetes Research Foundation Chennai. Food (Nutrilab, Bangalore) and water were given *ad libitum* to the animals. High fat diet (57%) was procured from the National Institute of Nutrition, Hyderabad, India. After acclimatization, basal fasting plasma/serum biochemical markers were estimated. Mice were randomly divided into two groups *i*.*e*., mice fed with normal pellet diet (NPD; n = 5) and mice fed with high fat diet (HFD; n = 6), with similar average body weight and plasma glucose on day 0. All groups were fed with respective diet and water *ad libitum* for 6 months. All biochemical and preclinical measurements were done as per the standard procedures (S2 Table). At the end of the protocol, HFD fed animals were characterized as glucose-intolerant and insulin-resistant by OGTT (Oral Glucose Tolerance Test) and ITT (Insulin Tolerance Test), respectively. Serum samples (0.25 ml) were used to quantify candidate miRNA by qRT-PCR as previously described.

## Results

In this study, we have analysed the expression of circulating microRNAs in serum of 145 non obese subjects (50% males and 50% females) suffering from T2DM (n = 49) compared with controls (n = 49) or pre-diabetic subjects (n = 47). As shown on Table 1, both prediabetics (IGT) and patients with type 2 diabetes (T2DM) had poor glycemic control as reflected by significant higher fasting plasma glucose and 2 hr plasma glucose and higher HbA_1c_ values compared with controls. Subjects with prediabetes and patients with type 2 diabetes exhibited significantly higher insulin resistance as revealed by HOMA-IR estimation. The lipid levels and blood pressure did not significantly differ among the 3 groups.

### Profiling of Circulating miRNA

Serum miRNA profiling was first performed on a subgroup of 36 subjects from the 145 subjects (50% males and 50% females). T2DM and IGT patients of this identification sample only differed clinically from control subjects in fasting glucose and 2 hr plasma glucose and in fasting insulin. Both T2DM and IGT patients had significantly higher waist circumference than the control group of subjects (Table 1). The global serum miRNA profiling (miRCURY LNA microRNA Human panel I V3-Exiqon) detected an average of ~159 miRNAs per sample of which 112 miRNAs were detected in all groups. As shown on S1 Fig, the majority of the 112 miRNAs were expressed in a similar way and hierarchical clustering of the data did not permit to discriminate between the 3 groups. Statistical data analysis revealed that the mean expression level of 9 miRNAs (*i*.*e*.; miR-128, miR-99b-5p, miR-130b-3p, miR-142-3p, miR-374a-5p, miR-423-5p, miR-484, miR-629-5p, let-7d-3p) was significantly different (student *t*-test *p*<0.05) across the studied groups (Table 2). Two miRNAs (*i*.*e*.; miR-128 and miR-99b-5p) had increased circulating concentrations both in prediabetic subjects and patients with type 2 diabetes compared to control subjects (Table 2).

**Table 2:** List of differentially expressed circulating miRNAs in the identification group.

Groups	miRNAs	Fold changes	*p* value
** **	** **	** **	** **
**NGT vs IGT**	**hsa-miR-128**	**1.42**	**0.041**
** **	**hsa-miR-99b-5p**	**1.41**	**0.028**
** **	** **	** **	** **
**IGT vs T2DM**	**hsa-miR-130b-3p**	**1.41**	**0.035**
** **	**hsa-miR-142-3p**	**0.57**	**0.0041**
** **	**hsa-miR-374a-5p**	**0.58**	**0.039**
** **	**hsa-miR-423-5p**	**1.54**	**0.011**
** **	**hsa-miR-484**	**1.37**	**0.004**
** **	**hsa-miR-629-5p**	**1.84**	**0.030**
** **	** **	** **	** **
**NGT vs T2DM**	**hsa-let-7d-3p**	**1.26**	**0.029**
** **	**hsa-miR-128**	**1.50**	**0.047**
** **	**hsa-miR-130b-3p**	**1.46**	**0.049**
** **	**hsa-miR-142-3p**	**0.65**	**0.039**

Ct values were used to evaluate differences among the identification group and to select candidate miRNAs

**Fig 1:**
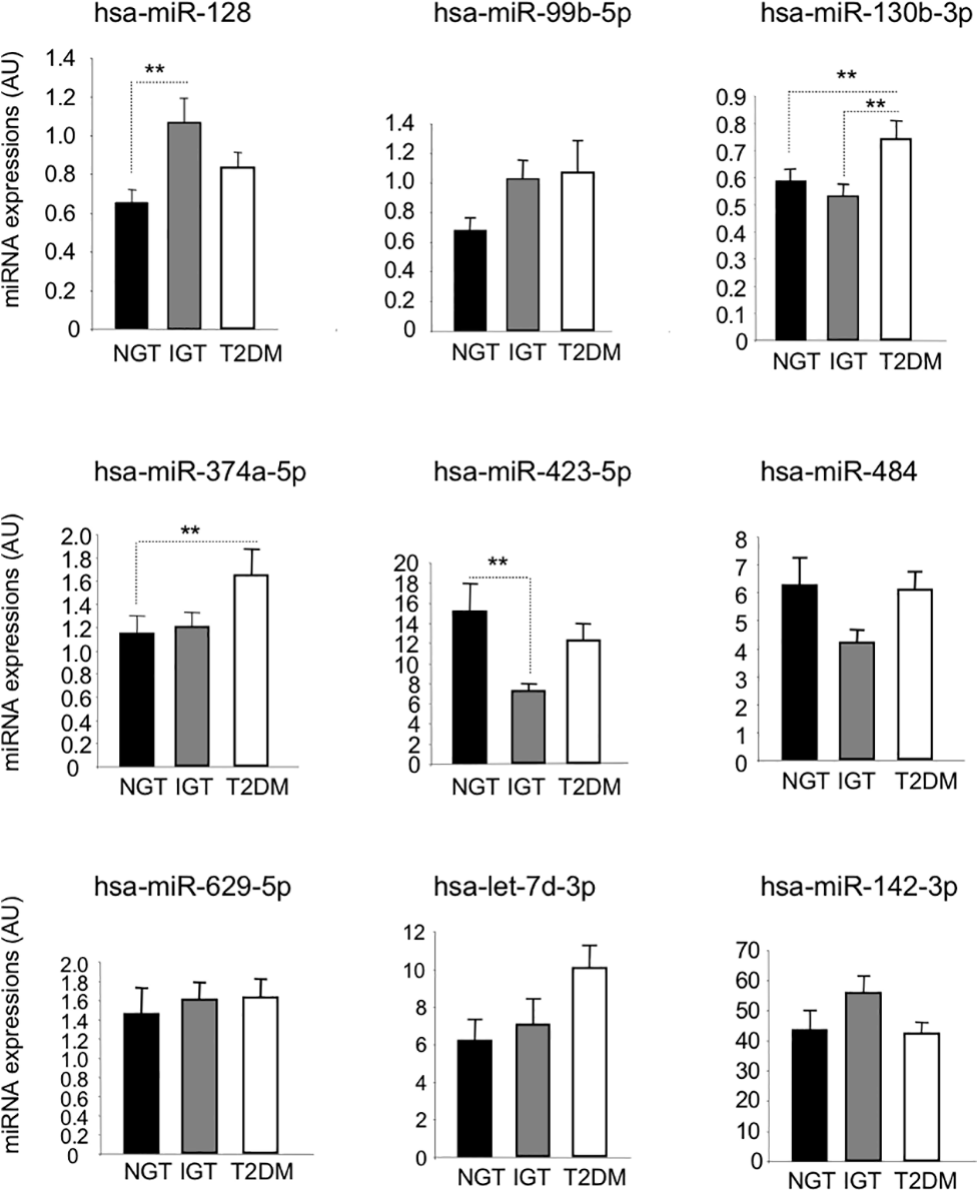
Circulating miRNA concentration of the 9 selected miRNAs, in the whole cohort of 145 individuals. ** = *p* values<0.05. Data are expressed as arbitrary units (AU).

All the 9 miRNAs differentially expressed among the discovery groups were analysed by quantitative RT-PCR using the whole cohort of 145 individuals (Fig 1) containing 50% men and 50% women. As shown on Fig 1, altered expressions of miR-128 and miR-423-5p in pre-diabetic patients compared to controls were confirmed with high significance (p<0.05). In addition, miR-374a-5p and miR-130b-3p were altered in the same direction in the whole group of patients suffering from T2DM than in discovery group, compared to the control group of subjects, and miR-130b-3p was only affected in the T2DM group. Altered expressions of miR-629a-5p, let-7d-3p, miR-142-3p and miR-484 were not confirmed in the whole cohort (*p*<0.05).

**Fig 2:**
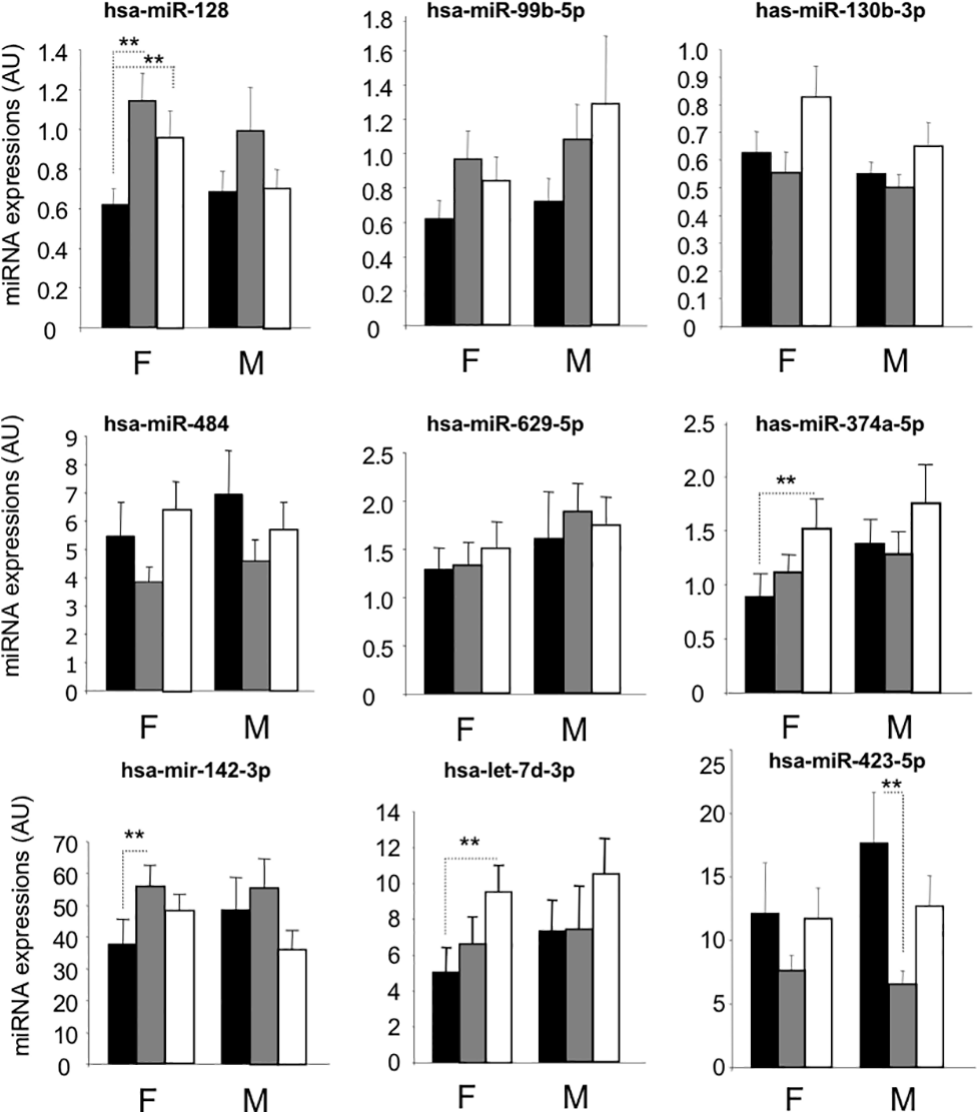
Circulating miRNA concentrations of the 9 selected miRNAs, in the whole cohort of 145 individuals, but considering men (M) and women (F) independently. ** = *p* values<0.05. Data are expressed as arbitrary units (AU). Black, control subjects; Grey, pre-diabetic subjects; White, diabetic patients.

Then miRNA expressions were analysed in women and men independently (Fig 2). Altered expressions of miR-128 in pre-diabetic state *vs* control was found significant in the group of women only. On the contrary, altered expression of miR-423-5p was significantly decreased in the group of men (NGT *vs* IGT) only. The increase level of miR-374a-5p in the diabetic subjects *vs* controls was found significant in the group of women only. Finally, miR-142-3p which was not differentially expressed considering the whole cohort (Fig 1), was found differently expressed when the group of women was analysed independently (NGT *vs* IGT, Fig 2).

### Correlation of miRNAs with Metabolic Parameters

Correlations between the levels of the validated miRNAs with the metabolic parameters were calculated in the whole cohort of subjects (Table 3). They were positively or negatively correlated to cholesterol levels, HbA_1C_, insulin resistance and hyperinsulinemia. MiR-128, which was increased in prediabetic patients (Fig 1), was positively correlated with serum cholesterol. On the contrary, miR-423-5p decreased in the serum of prediabetic patients (Fig 1) was negatively correlated to HDL-cholesterol.

**Table 3:** Correlations between circulating miRNA concentrations and metabolic parameters in the study subjects.

	miRNAs	Metabolic parameters	Estimate	StdErr	tValue	Probt
Correlations not adjusted for sex	miR-128	Serum-cholesterol	0.003482	0.001333	2.61	0.0099
					
	miR-423-5p	HDL-cholesterol	-0.3067	0.1363	-2.25	0.0260
					
	miR-130b-3p	HbA1C	0.1902	0.04745	4.01	<.0001
	miR-374a-5p	HbA1C	0.1745	0.07149	2.44	0.0159
					
	miR-374a-5p	HOMA-IR	0.1093	0.04364	2.50	0.0134
					
	miR-374a-5p	Fasting-Insulin	0.04148	0.01844	2.25	0.0260
					
	miR-128	Serum-cholesterol	0.003594	0.001323	2.72	0.0074
					
	miR-423-5p	HDL-cholesterol	-0.3052	0.1266	-2.41	0.0172
					
Correlations adjusted for sex	miR-130b-3p	HbA1C	0.1903	0.04728	4.03	<.0001
	miR-374a-5p	HbA1C	0.1763	0.07174	2.46	0.0152
					
	miR-374a-5p	HOMA-IR	0.1082	0.04383	2.47	0.0147
					
	miR-374a-5p	Fasting-Insulin	0.04138	0.01852	2.23	0.0270
					

To calculate correlations, *Ct* values were converted into copy numbers (10^((Ct-25)/-3,3)*100) to take into account the logarithmic scale of the data.

### miRNA Target Genes Analysis

**Fig 3:**
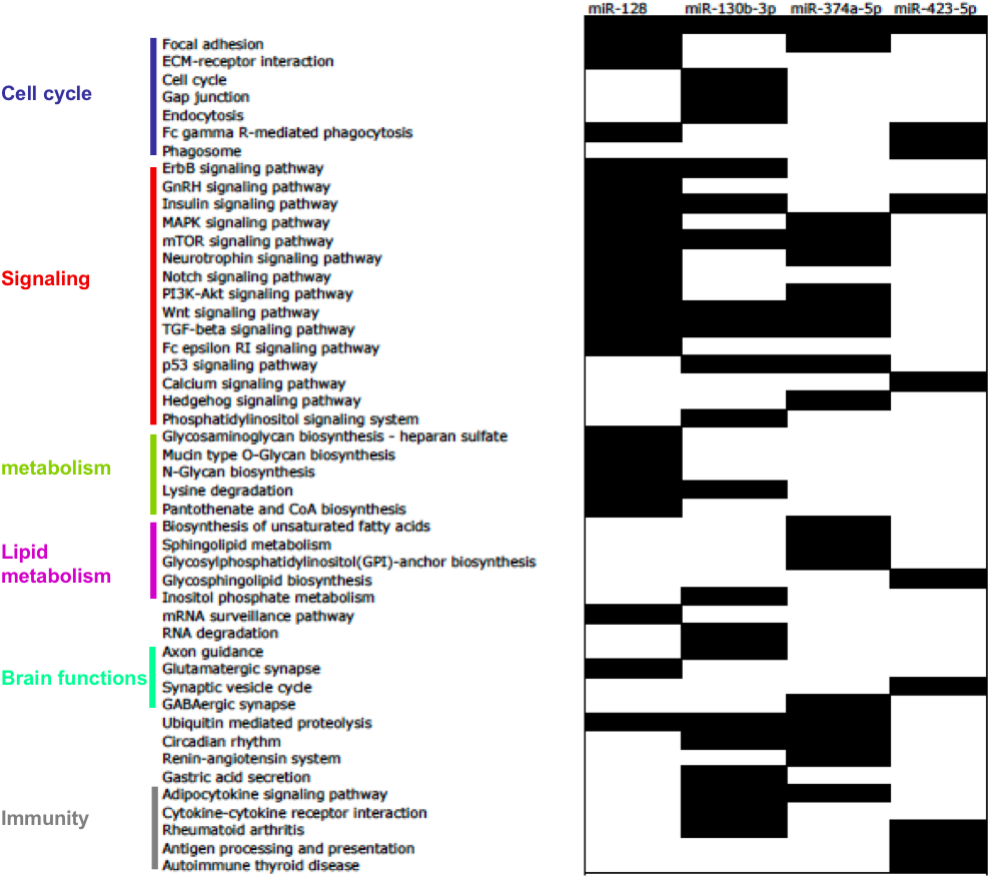
Predicted cellular pathways of the target genes of the significantly altered circulating miRNAs in serum of pre- and/or diabetic patients *vs* controls. Only validated miRNAs in the whole cohort were considered.

Target genes of the 4 validated miRNAs were predicted and significant cellular pathways affected by these genes were retrieved for each miRNA. Significant KEGG pathways are shown on Fig 3. There are related to cell cycle, signaling pathways, lipid metabolism, glycan biosynthesis, brain functions and immune system.

### Circulatory miRNA levels in HFD mice *vs* NPD fed mice

**Fig 4:**
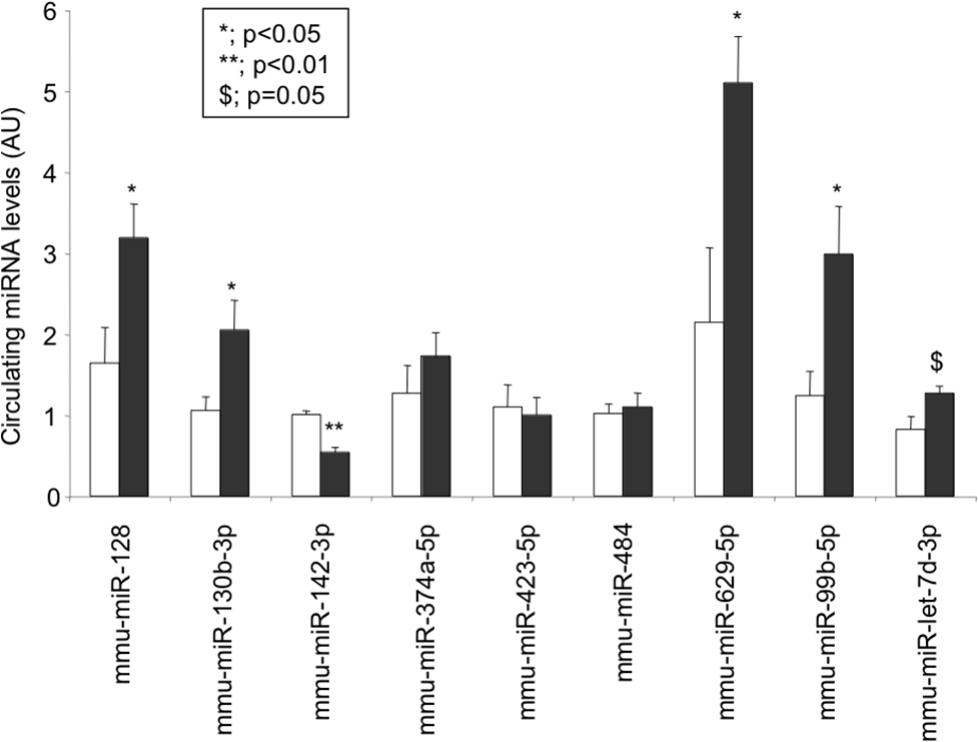
Circulating miRNA concentrations of the 9 selected miRNAs identified in the discovery group quantified in the serum of mice fed with normal pellet diet (NPD; n = 5) or with high fat diet (HFD; n = 6). Data are expressed as arbitrary units (AU). White, NPD mice; Black, HFD.

To further corroborate the associations assessed in human, the present study also analyzed by means of qRT-PCR circulating miRNA concentrations in the plasma of diet-induced diabetic mice (HFD). In the context of the well-known insulin-resistance effects, high-fat diet led to increased circulating concentrations of miR-128, miR-130b-3p, miR-99b-5p, miR-629a-5p and miR-let-7d-3p expression in HFD mice compared with NPD fed mice (p<0.05). Conversely, miR-142-3p was significantly lower in HFD animals compared to control mice (Fig 4) Correlations between the expressions of altered miRs (HFD *vs* NPD) with the metabolic parameters were calculated and are shown in Table 4. As in human pre-diabetic subjects, miR-128 was positively correlated with cholesterol.

**Table 4:** Correlations between circulating miRNA concentrations and metabolic parameters in mice.

miRNAs	metabolic parameters	Estimate	StdErr	tValue	Probt
miR-128	cholesterol	-0.0292	0.005164	-5.65	0.0005
miR-484	Glucose	-0.01151	0.00478	-2.41	0.04
miR-629-5p	Glucose	0,04474	0,01286	3,48	0,007
miR-128	Glucose	0,02141	0,00837	2,56	0,031
miR-130b-3p	HbA1c	-1.2578	0.4202	-2.99	0.019
miR-99b-5p	HbA1c	-2.4481	0.463	-5.29	0.001
miR-130b-3p	HDL	-0.03028	0.00876	-3.46	0.011
miR-99b-5p	HOMA_IR	0.1122	0.04224	2.66	0.026
miR-let-7d-3p	HOMA_IR	0.02725	0.01108	2.46	0.036
miR-99b-5p	TGL	0.02065	0.00898	2.3	0.047
miR-let-7d-3p	TGL	0.005752	0.00214	2.69	0.025
miR-99b-5p	VLDL	0.1033	0.04491	2.3	0.047
miR-let-7d-3p	VLDL	0.02876	0.0107	2.69	0.025

## Discussion

South Asians are characterized by a unique metabolic profile with higher insulin levels [3], a greater degree of insulin resistance [4], greater abdominal adiposity *i*.*e*., higher waist circumference despite lower body mass index [2] and a higher prevalence of diabetes [5]. Insulin resistance has been demonstrated in Asian Indians even during adolescence [6] and hyperinsulinemia seems to be present in Asian Indians even at birth [7]. It appears that some of the increased propensity for South Asians to develop insulin resistance could be attributable to greater accumulation of visceral fat [35, 36]. Thus South Asians are at elevated risk for T2DM, compared with Caucasian and other ethnic groups [2]. As a consequence, circulating miRNAs previously identified in caucasian diabetic population [26, 28, 33, 37] might not be relevant for Asian population. Thus, in this study, we have analysed the expression of circulating miRNAs in serum of non obese Indian subjects suffering from T2DM compared with controls or pre-diabetic subjects. Considering the whole population, this analysis revealed 4 differentially expressed miRNAs (miR-128, miR-130b-3p, miR-374a-5p, miR-423-5p) in subjects with prediabetes and T2DM patients compared to control subjects with normal glucose tolerance. Our study also highlighted that some miRNAs (miR-128 and miR-374a, miR-142-3p, let-7d-3p, miR-423-5p) had sex-specific associations with prediabetes or diabetes. For example, the expression of miR-128, which was stronly correlated with the level of cholesterol, was significantly more increased in the serum of pre- and diabetic women and in men. In fact, for the same BMI and waist circumferences, we found that the groups of control-, pre-diabetic- and diabetic women enrolled in this study, had significantly more cholesterol than the group of control, pre-diabetic and diabetic men. In addition, the group of diabetic men was significantly younger than the group of diabetic women (41.7 ± 1.45 vs 47.08 ± 1.72; *p* = 0.02) which could explain that some miRNAs differentially expressed between controls and diabetics were not significantly altered in the group of men (*i*.*e*.; miR-128; miR-374a, miR-142-3p and let-7d-3p). However, whatever the considered miRNAs, their variations among the groups were in the same direction for both men and women (Fig 2).

Among the altered circulating miRNAs identified in this study, miR-128 has never been described in previous studies [26–33, 37]. In our study, circulatory miR-128 level was found increased in prediabetic subjects and was confirmed in diet-induced diabetic mice compared to controls. Interestingly, miR-128 which was positively correlated with cholesterol level in our Indian population and in the diabetic mice, has been shown to post-transcriptionally inhibit the cholesterol transporters and play a regulatory role in cholesterol efflux and cholesterol homeotasis [38]. Moreover, it was demonstrated that miR-128 down-regulated genes involved in insulin signaling (*e*.*g*.; insulin receptor, insulin receptor substrate-1 and phosphatidylinositol 3-kinases regulatory 1) in muscle cells [39]. Taking into consideration of all these data, we suggested that the increased level of circulating miR-128 might be linked to the development of dyslipedemia associated with T2DM.

Beside its potential predictive value, the profile of circulating miRNAs could also furnish precious information about the pathophysiology of the disease. Although the precise source of blood miRNAs is presently unknown, most of them can be produced by a variety of cells, including all organs whose functions are altered in T2DM (*e*.*g*.; pancreas, liver, adipose tissue and skaletal muscle). In agreement, the analysis of the biological functions of the target genes for the 4 differentially expressed miRNAs in pre- and or diabetic subjects, revealed their roles in signaling pathways (*e*.*g*.; insulin and PI3K-AKT signaling pathways), cell proliferation, lipid and glycan metabolism, adipocytokine signaling and immune response. All these functions are altered in the insulin sensitive tissues of prediabetes and T2DM. More interestingly, this analysis also showed that some of the differentially expressed miRNA target genes were predicted to regulate brain functions. Recent studies support the concept that T2DM is associated with cognitive dysfunctions and structural brain changes [40]. Indeed, it is well-admitted that brain plays a key role in normal glucose regulation and in T2DM development and there is evidence for a brain-centered system that can lower blood glucose via insulin and non-insulin dependent mechanisms [41]. Among the 4 differentially expressed miRNAs of this study, miR-128 is a brain enriched miRNA which is highly expressed during neuronal differentiation [42]. It governs neuronal excitability and motor behavior in mice [43]. Interestingly, miR-128 has been shown to be one of the circulatory miRNA biomarkers for detection of mild cognitive impairment [44]. Recently, GWAS (Genome Wide Association Study) for type 2 diabetes in Indians have identified a new susceptibility locus which harbors the genes that are involved in neurological processes further suggesting a neurological component in the etiology of T2DM [45]. Therefore, alteration of circulating miRNA in T2DM might highlight cognitive dysfunctions associated with metabolic diseases.

To conclude, our study is the first of its kind in India to demonstrate altered levels of serum miRNAs in subjects with IGT and T2DM patients compared to control subjects. Among the altered miRNAs identified in this study, miR-128 has never been described in previous studies/populations and deserves further in-depth investigations. Unlike GWAS which has limited clinical intrepretation, circulatory miRNAs might be clinically relevant robust biomarkers. Our findings imply directionality towards biomarker potential of miRNAs in the prevention/diagnosis/treatment outcomes of diabetes.

## Supporting Information

S1 Fig Cluster and Tree View softwares (ref) were used for hierarchical clustering of normalized miRNA expressions of the 112 miRNA expressed in all subjects (discovery group). ref = Eisen, M.B., Spellman, P.T., Brown, P.O., and Botstein, D. 1998. Cluster Analysis and Display of Genome-Wide Expression Patterns. Proc. Natl. Acad. Sci. U S A. 95: 14863–14868.
(TIFF)

S1 Table Metabolic parameters significantly different between men and women in each group (NGT, IGT and T2DM). p values<0.05 (*) are significant, F = female and M = male
(XLS)

S2 Table Metabolic parameters of mice fed with normal pellet diet (NPD group) or with high fat diet (HFD group) for 6 months. ND = not determined; in black bold = significant ‘p’ values
(XLS)
