# Supplementary figures and images for: Circulating MiRNAs of ‘Asian Indian Phenotype’ Identified in Subjects with Impaired Glucose Tolerance and Patients with Type 2 Diabetes
Source: PLoS One. 2015 May 28;10(5):e0128372. doi: 10.1371/journal.pone.0128372 (PMC4447457; doi:10.1371/journal.pone.0128372)

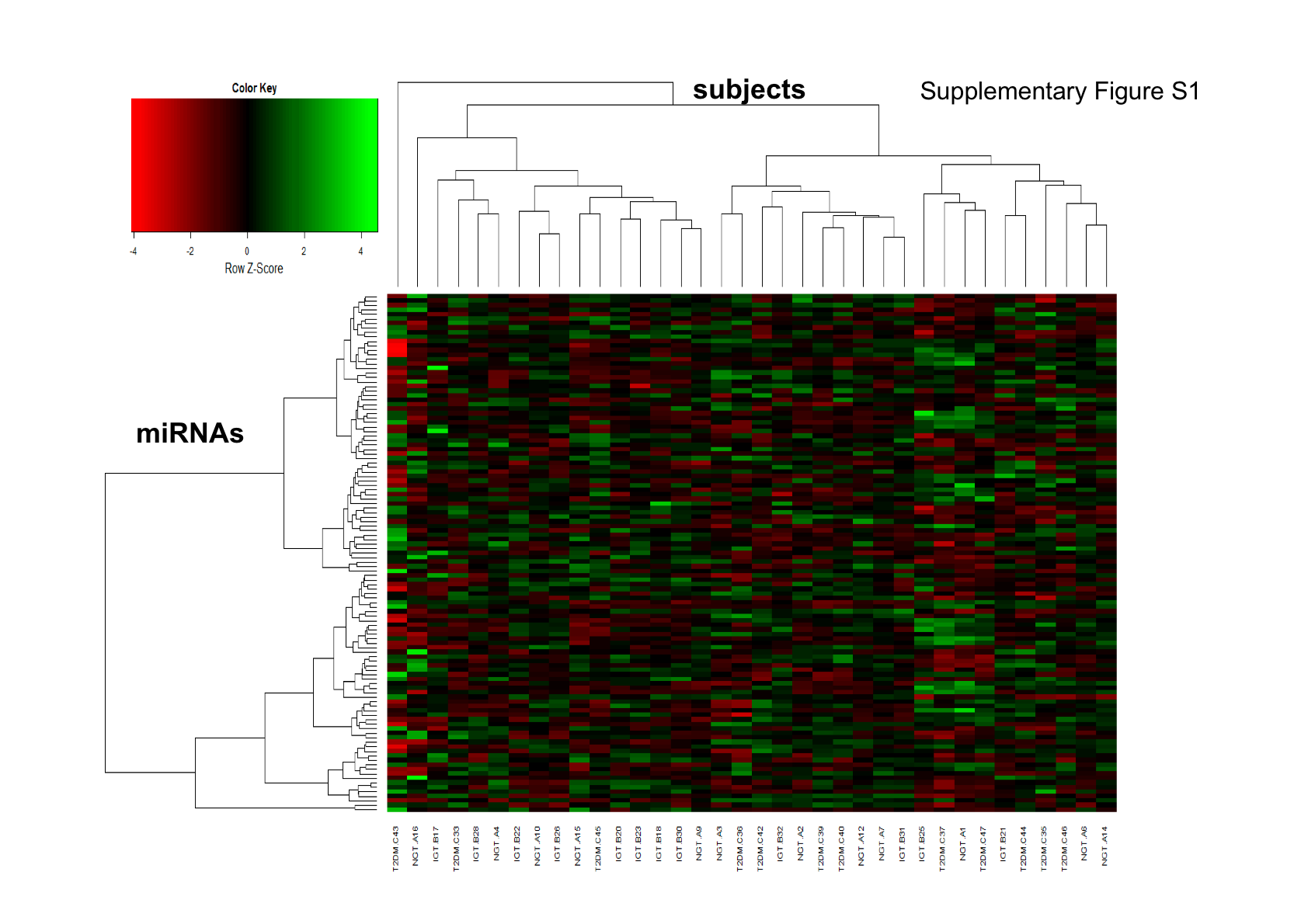

Supplement: S1 Fig — ref = Eisen, M.B., Spellman, P.T., Brown, P.O., and Botstein, D. 1998. Cluster Analysis and Display of Genome-Wide Expression Patterns. Proc. Natl. Acad. Sci. U S A. 95: 14863–14868. (TIFF) [file pone.0128372.s001.tiff]
